# The Role of Astrocytes in CNS Disorders: Historic and Contemporary Views

**DOI:** 10.3390/cells13161388

**Published:** 2024-08-20

**Authors:** Michael Brenner, Vladimir Parpura

**Affiliations:** 1 Department of Neurobiology, The University of Alabama at Birmingham, Birmingham, AL 35294, USA; 2 International Translational Neuroscience Research Institute, Zhejiang Chinese Medical University, Hangzhou 310053, China

## 1. Introduction

This Special Issue of *Cells* presents a collection of 22 published, peer-reviewed articles on the theme of “Astrocytes in CNS Disorders,” including 9 reviews of the evidence implicating astrocytes in the etiology of specific disorders, and 13 original research papers providing such evidence. As discussed in these papers, astrocyte dysfunction is now implicated in disorders once thought to be solely of neuronal origin, such as epilepsy [1,2] and amyotrophic lateral sclerosis [3]. It is also implicated in disorders previously ascribed solely to oligodendrocytes, like vanishing white matter disease [4] and megalencephalic leukoencephalopathy with subcortical cysts [5]. In some instances, the cause of the disorder is a defect originating in astrocytes. The first and perhaps most definitive example is Alexander disease, in which coding mutations in the *GFAP* gene cause massive dysmyelination and perturb multiple neuronal circuits. In other instances, induced disruption of normal astrocytic functions, such as glutamate transport or potassium flux, exacerbates the disorder. Although the identification of specific astrocyte-mediated mechanisms contributing to disorders as described in these papers is a relatively new endeavor, the likelihood that astrocytes have a role in CNS disorders was recognized by the earliest investigators of this cell type. Herein, we briefly review this historical context, followed by introductions to the current contributions.

## 2. A Brief History of Astrocytes and Their Role in CNS Disorders

Astrocytes are a molecularly and morphologically diverse subtype of neuroglia in the central nervous system (CNS) [6]. The first published observation of neuroglia was in 1858 by Rudolf Ludwig Karl Virchow [7], a German physician and pathologist, who noted the mass present between neurons and referred to it as “neuroglia” (*nervenkitt*). Virchow considered this mass to be acellular, serving to hold neurons in place. A cellular component of neuroglia was recognized in 1872, by Moritz Jastrowitz, a German internist and psychiatrist, who described spider-like glial cells (*spinnenähnliche gliazellen*) [8]. In 1893, British pathologist William Lloyd Andriezen and Swiss anatomist Albert von Kölliker subdivided neuroglia (presumably astrocytes) based on their morphology [9,10]. Andriezen described *fibrous* cells as having processes that are “extremely long, smooth contoured, and of uniform calibre”; whereas *protoplasmic* glial cell processes are much shorter in length, “exhibit great variations of calibre” and have the “striking feature” of a “shaggy granular contour.” This latter feature likely equates to the recently described “bushy” morphology of astrocytes (e.g., ref. [11]). Andriezen also observed that the fibrous cells tended to be present in white matter, whereas the protoplasmic cells were generally present in gray matter. Shortly thereafter, in 1895, Hungarian anatomist and histologist Michael von Lenhossék coined the term “astrocytes” (*astrocyten*) for these stellate neuroglia; the term was rooted in the ancient Greek words *astron* (star) and *kytos* (container) [12].

That astrocytes, like neurons, derive from the neuroectoderm was demonstrated in 1889 by Wilhelm His, a Swiss-born anatomist and cardiologist, and the inventor of a microtome [13,14]. Study of astrocytes was facilitated by development of a specific stain in 1913 by Santiago Ramón y Cajal, a Spanish pathologist and histologist with a keen interest in the CNS [15]. We know today that his gold sublimation technique targets the astrocyte cytoskeleton, in particular, intermediate filaments, the major component of which is glial fibrillary acidic protein (GFAP), a commonly used astrocyte marker [16]. The gold sublimate method is tedious (a recipe is available on pp. 323–324 of ref. [17]) and has been replaced by the simpler and more reproducible method of immunostaining against GFAP (e.g., ref. [18]). Nonetheless, by using the gold sublimate method to analyze the adult human hippocampus, Cajal inferred a proliferating ability of adult astrocytes, as he observed “*astrocitos gemelos*”, which translates to twin astrocytes; i.e., pairs of astrocytes held together by their somata [15].

As morphological studies advanced in the late 19th century and the beginning of the 20th century, speculations of astrocyte function also emerged. Camillo Golgi, an Italian biologist and pathologist, speculated in 1895 that they serve “for distributing the nutritive material”; i.e., they have a metabolic role [19]. Ernesto Lugaro, an Italian psychiatrist, expanded on this idea in 1907, suggesting that astrocytes may take up and metabolize neuroactive substances at the synapse. He wrote:

“Elsewhere I have exposed the arguments that can make us to think that the actions carried out at the level of the neuronal articulations [synapses], between neuronal terminals and dendrites and cellular bodies of subsequent neurons, are of chemical nature. Every nervous ending would undergo in the moment of the excitation a chemical modification and this chemical modification would act as a stimulus on the other neuron. If that were [true], the interneuronal articulations would be a center of active chemical exchanges; and one would therefore comprise the infiltrating of protoplasmic “tufted” extensions of neuroglia in all the nearby free interstices, in order to perhaps pick and instantaneously to fix even the smallest product of refusal” (page 294 of ref. [20]; translation in [21]).

Experimental confirmation of Lugaro’s speculation awaited an elegant study performed a century later demonstrating that astrocytes in culture responded to glutamatergic neurotransmission by uptake of glutamate via their plasma membrane glutamate transporters, a process which contributes to the termination of synaptic events [22]. We know today that 80% of glutamate removal from the extracellular space in the CNS is done by astrocytes, while the remaining 20% is taken up by neurons [23]. A metabolic cross-talk between astrocytes and neurons was also discovered, whereby astrocytes convert glutamate to glutamine [24], which is then released to the extracellular space to be taken up by neurons, which convert the glutamine to glutamate and GABA. Furthermore, Lugaro’s concept that three synaptic compartments (pre- and post-synaptic neuronal elements as well as the glial element) constitute a functional synapse found further theoretical traction in the so-called *triad* [25]. The concept of the “tripartite synapse” expanded the synaptic role of astrocytes from serving solely for glutamate removal to being an active participant by the finding that “gliotransmission”; e.g., glutamate released from astrocytes in a Ca^2+^-dependent manner [26], can modulate synaptic activity [27,28].

Although discovery of Ca^2+^-dependent glutamate release from astrocytes is a relatively new finding, the possibility that astrocytes had secretory properties was raised over a hundred years ago by Hans Held in Germany and Jean Nageotte in France (for a review, see [29]). Held observed dark molybdenum-hematoxylin-stained granular inclusions in processes of marginal subpial astrocytes in 1909 [30]. Cytosolic granules in glial cells were also reported by several microanatomists of that era (1906–1916), including Alzheimer, Eisath, Fieandt, Cajal, Achucarro and Rio Hortega; with Alzheimer hypothesizing that these granules are evidence for glial secretion mediating metabolic support for neurons (for a review and references, see [29]). In 1910, Nageotte provided the first spatio-temporal description of secretion from gray matter neuroglia, which were likely astrocytes. It should be noted, however, that he misidentified the secretory granules as mitochondrial fragments. He wrote (pages 1068–1069 of ref. [31], translated by Vedrana Montana, Zhejiang Chinese Medical University and Mathieu Lesort, The University of Alabama at Birmingham, with the parenthetical addition for clarification):

“The facts that I have just observed seem to shed new light on the physiology of neuroglial cells, not only of those which associate closely to neuronal cells and deserve the name of satellite cells, but also, and especially, of cells which are in connection with the vasculature walls.

Indeed, I was able to present evidence of robust active secretion phenomena in the protoplasm of these cells in rabbit and guinea pig. This observation is especially visible within the protoplasmic extensions which cross the empty space created by the retraction of tissues around the vascular walls, on which they [neuroglial cell extensions] attach using an enlarged foot.

In a previous note, I have described the mitochondria that exist in these protoplasmic extensions, and I have shown that many, and maybe all of the granulations located in the gray matter outside the protoplasm of neuronal cells, in reality belong to the neuroglia. Today, I am poised to follow the evolution that occurs within these granulations and to show their progressive transformation into secretion grains. These phenomena are exactly similar to those described by Altmann in the glandular cells; the observed granulations are of three types: 1. excessively small round grains that, by the Altmann method, color themselves in intense red; 2. more voluminous grains, with clear centers; 3. grains that do not take color with fuchsin. The last ones are slightly smaller than the most voluminous red grains. All intermediates exist between these three types, which represent the successive phases of the transformation of mitochondria into secretion grains.

Using Benda’s method, the smallest grains color themselves in blue, the largest ones in purple and the secretion grains in red.”

More modern and direct evidence for astrocyte transmitter release came from demonstration that primary cultured astrocytes can release taurine [32]. We know today that astrocytes can release many chemical transmitters, neuromodulators, hormones, and metabolic, trophic and plastic factors, under various conditions and by several different mechanisms (reviewed in [33]). For instance, glutamate can be released from astrocytes: (i) through plasmalemmal channels like anion channels [34], hemichannels formed from unpaired connexons [35], or pore-forming P2X_7_ ionotropic purinergic receptors [36]; (ii) through transporters, such as the reversal of uptake via plasma membrane excitatory amino acid transporters [37], exchange via the cystine-glutamate antiporter [38] or through organic anion transporters [39]; and (iii) via Ca^2+^-dependent exocytosis [26].

That dynamic neuronal-glial interactions underlie brain functions was postulated as long ago as the late 19th century. Carl Ludwig Schleich, a German surgeon, suggested that variations in the volume of glial cells represented the mechanism for control of neuronal communication; swollen glial cells inhibited neuronal communication, and communication was facilitated when glia shrink [40]. Cajal suggested that astrocytes act as a switch between active and passive states in neuronal networks; retraction of astroglial processes allows information to flow during wakefulness, whereas expansion of astroglial processes disrupts interneuronal connectivity, thus inducing sleep [41,42]. Variants of these ideas have since been experimentally demonstrated; for example, in the supraoptic nucleus of the hypothalamus the retraction of astrocytic processes during lactation or chronic dehydration allows for an increased formation of dendrodendritic electrical synapses between magnocellular neurons, resulting in synchronization of neurons in discharging action potentials (reviewed in [43]).

From the above postulated and proven properties of astrocytes it was to be expected that their faulty functioning would contribute to or even cause CNS disorders. Indeed, they have been implicated in various CNS pathologies from the onset of their description. Virchow thought that neuroglia represent “one of the most frequent seats of morbid change” [7], while Andriezen considered astrocytes to “exhibit a morbid hypertrophy in pathological conditions” [9]. We now introduce the 22 papers in this Special Issue that fulfill this expectation.

## 3. Overview of the Special Issue Papers

Leukodystrophies are a group of heritable neurological diseases affecting primarily the white matter. Alexander disease (AxD) is a rare, often fatal leukodystrophy of humans, in which the primary deficit in astrocytes affects oligodendrocytes and neurons. Dominant gain-of-function mutations within the coding sequence of the *GFAP* gene are responsible for nearly all cases of Alexander disease [44]. Using molecular genetics and a pre-clinical mouse model of AxD, Hagemann et al. [45] demonstrate that signal transducer and activator of transcription (STAT)-3 in astrocytes plays a role in AxD pathology by promoting GFAP accumulation and aggregation, one of the hallmarks of AxD histopathology. Preventing STAT-3 activation could therefore be therapeutic for AxD in human patients.

Vanishing white matter (VWM) disease is another heritable and progressive leukodystrophy which mainly affects the CNS. In addition to loss of white matter, cortical atrophy is present in long-term patients. VWM is unusual in its progression, as stressors such as infection, mild head trauma or other injuries, can lead to periods of rapid and severe deterioration [46]. The genetic defect may occur in any of the five housekeeping genes that encode individual subunits of eukaryotic translation initiation factor eIF2B (*EIF2B1*, *EIF2B2*, *EIF2B3*, *EIF2B4* and *EIF2B5*) [47]. Surprisingly, although the defect is in a general housekeeping gene, oligodendrocytes and astrocytes are predominantly affected among all cell types. In this Special Issue, Man et al. [4] analyzed the cortex of the middle frontal gyrus of VWM patients for protein composition using mass spectrometry and for astrocyte morphology using immunochemistry. Of the several pathways identified by gene ontology analysis, 14 proteins, all downregulated in VWM, were associated with mitochondrial activity; 10 of the genes encoding these 14 proteins are expressed at least five times more strongly in astrocytes than in neurons or oligodendrocytes. Further implicating cortical astrocytes in VWM, they appeared immature, unreactive, and morphologically less complex than astrocytes from control patients. These results are the first description of molecular and pathological changes in the frontal cortex of VWM patients.

Spinocerebellar ataxia type 1 (SCA1) is an inherited progressive neurodegenerative disease. Its cause is expansion in the *ataxin1 (ATXN1*) gene of the triplet nucleotide repeat cytosine-adenine-guanine (CAG), which encodes glutamine. An expansion of the CAG tract to at least 39 repeats results in a fully penetrant pathogenic allele [48]. Normal ataxin-1 is a DNA-binding protein with unknown function. Expression of the ataxin-1 gene occurs in neurons, astrocytes and microglia. Both of the glial cell types become activated, likely by signals from distressed neurons [49], with reactive astrocytes having been shown to contribute to pathogenesis of SCA1 [50]. Using a knock-in *Atxn1*^154Q/2Q^ mouse model of SCA1, in this Special Issue Rosa et al. [51] report spatial and temporal variation in the reactivity of astrocytes and their expression of neuroprotective genes in the four clinically relevant brain regions: cerebellum, brainstem, hippocampus and motor cortex. Changes were observed early in the disease progression, before neuronal loss. Regional variability in astrocyte homeostatic functions correlated with subsequent pathology, implicating astrocytes in the region- and stage-specific pathogenesis of SCA1.

Glycogen is a metabolically dynamic polymer that serves as a storage form for glucose [52]. In the brain it is primarily located in astrocytes, where it is mobilized for such functions as memory formation [53], protection from hypoxia [54] and glutamatergic gliotransmission [55]. Three glycogen storage diseases affect the CNS: adult polyglucosan body disease, which generally presents in the fifth or sixth decade of life, and Lafora disease and polyglucosan body myopathy 1 with or without immunodeficiency, which occur in children. Each of these disorders is caused by accumulation of insufficiently branched glycogen, which becomes sequestered in aggregates referred to as Lafora bodies or *corpora amylacea*. Duran [56] reviews what is known about the genetic defect in each of these three disorders, and describes the experimental evidence for a primary role for astrocytes in disease development. Perhaps widening the relevance of these glycogen storage disorders, Duran points out that similar aggregates also accumulate in several other CNS diseases, such as Alzheimer’s and Parkinson’s diseases, and temporal lobe epilepsy.

A defining feature of Parkinson’s disease (PD) is the degeneration of dopaminergic neurons in the basal ganglia leading to movement disorders. Although variants of several genes have been associated with PD, about 90% of cases are idiopathic, suggesting involvement of environmental toxins. Nevertheless, studies of the genetic variants have been pursued with the expectation that they will provide clues to causative environmental factors. In this Special Issue, Kim et al. [57] review results from PD models induced by either genetic variants or neurotoxins that point to astrocyte dysfunction as contributing to dopaminergic neuron loss. Greater attention to this role of astrocytes in PD may lead to development of more effective therapeutics.

Alzheimer’s disease (AD) is characterized by a progressive brain parenchyma atrophy leading to severe dementia. At the histopathological level there is a presence of both senile plaques, which consist of extracellular deposits of β-amyloid protein, and intra-neuronal accumulation of neurofibrillary tangles, which are composed of tau-protein filaments [58,59]. Astrocyte involvement in AD is suggested by studies in transgenic animal models that find that astrocytes become atrophic at the very early stages of pathology and then become reactive prior to the formation of plaques [60,61,62]. It is also suggested by AD model mice having enhanced Ca^2+^ signaling. That this enhanced signaling can be induced by neuronally-derived soluble Aβ injected into wild type mice in the absence of plaques suggests that it is an early event in AD development [63]. In this Special Issue, Fontana et al. [64] review and correlate results from clinical positron emission tomography (PET) analyses that detect astrocyte reactivity, the presence of Aβ plaques and tau tangles. These findings were also correlated with those for cerebrospinal fluid (CSF) and blood markers of astrogliosis and AD. The authors observe two waves of astrogliosis; the first is associated with an early increase in monoamine oxidase B that precedes Aβ plaque formation by up to 10 years, and the second is associated with a later increase in GFAP, which correlates with plaque deposition. These observations raise the possibility that the early activation phase of astrocytes may contribute to the progression of AD. They also raise the promise that monitoring markers of early astrogliosis may permit detection of AD in its very early stages.

Like AD, amyotrophic lateral sclerosis (ALS, also known as Lou Gehrig’s disease) is a progressive, fatal neurological disorder involving the death of neurons—in this case, motor neurons. In addition to motor deficits, about 15% of ALS patients develop behavioral and cognitive disorders, leading to co-diagnosis of frontotemporal dementia (FTD). Conversely, about 20% of FTD patients are co-diagnosed with ALS. In this Special Issue Valori et al. [3] review data demonstrating astrocyte reactivity as an early event in both ALS and FTD, with the resulting alteration of astrocyte functions perhaps contributing to disease progression. Data reviewed include transcriptome analyses of patients and of mouse models, and detection of astrocyte reactivity by PET imaging and fluid biomarkers, similar to those described in the above-mentioned Fontana et al. [64] AD review. Like for AD, appreciation of the early role of astrocytes in ALS and FTD may facilitate early diagnosis and be the basis for therapeutic intervention.

Two papers in this Special Issue explore the role of astrocytes in neurotrauma—injury to the CNS resulting from an abrupt external physical assault. Tissue directly impacted by traumatic brain injury (TBI) is irretrievably lost, and adjacent (penumbral) tissue is at risk due to hypoperfusion, excitotoxicity, neuroinflammation and edema. Saving this penumbral tissue is thus the focus of medical intervention. Astrocytes respond to neurotrauma by reactive astrogliosis that has both detrimental and supportive effects on the outcome of TBI. In this Special Issue, Michinaga et al. [65] review the primacy of the astrocyte endothelin receptor B (ET_B_-R) in these astrocyte responses. Levels of both astrocytic ET_B_-R and endothelin-1 secretion are increased directly by TBI by an unknown mechanism. The resultant activation of the ET_B_-R triggers astrocyte reactivity, leading to secretion of cytokines and proteinases that disrupt the blood-brain barrier (BBB), contributing to both edema and neuroinflammation in the acute stage of injury. Studies in animal models suggest that drugs inhibiting the ET_B_-R may be therapeutic in the early stage of injury. However, in the recovery stage of TBI, the astrocytic ET_B_-R mediates release of neurotrophic factors that promote repair of the damaged brain parenchyma. Thus, in this later phase of the injury process, stimulation of ET_B_-R activity may be therapeutic.

As mentioned above, excitotoxicity is one of the pathological processes detrimental to the penumbra following TBI. A major contributing factor is the downregulation of glutamate uptake in reactive astrocytes [66]. Prevention of such loss could thus have therapeutic value. In this Special Issue, Gržeta Krpan et al. [67] use an in vitro simulated (by stretch) TBI model to demonstrate that colloidal single-walled carbon nanotubes derivatized with polyethylene glycol can prevent the injury-induced reduction in the level of plasmalemmal excitatory amino acid transporter 1 in astrocytes. A possible additional therapeutic benefit of this nanomaterial is that it caused an increase in the release of cytokines from injured astrocytes that were mainly anti-inflammatory. These might ameliorate the effects of the excess of proinflammatory cytokines present in TBI that contribute to disruption of the BBB, brain edema and neuroinflammation.

Other papers in this Special Issue focus on mechanisms of edema in CNS injury. Garcia et al. [68] review experimental evidence for a role for aquaporin 4 (APQ-4) in spinal cord injury. Particularly relevant to causality are studies performed with general and astrocyte-specific AQP-4 knockout mice. Following an injury that disrupts the blood-spinal cord barrier (BSCB), and thus produces vasogenic edema, the AQP-4 knockout mice are compromised in resolution of this edema. In contrast, following an injury producing cytotoxic edema, in which there is no compromise of the BSCB but swelling occurs in astrocytes due to water uptake, edema and its sequelae are ameliorated in the AQP-4 knockout mice. These results can be rationalized by the function of AQP-4 as a water channel. In cytotoxic edema, water enters astrocytes through the channel, and thus its absence attenuates the edema. In vasogenic edema, the compromised BSCB allows water to freely flow into the parenchyma along with proteins and other solutes, but once the BSCB heals, AQP-4 provides the channel for dissipating the edema. The insights provided by this review suggest that matching the timing and direction of AQP-4 manipulation to the type of traumatic CNS injury may provide clinical benefit.

Cytotoxic edema is counteracted by activation of volume-regulated anion channels (VRAC), whose release of chloride anion from the cell provides a driving force for export of water through the AQP-4 channels. In their research paper in this Special Issue, Brignone et al. [5] investigate the mechanism linking cell swelling to VRAC activation by building on observations from patients and animal models of megalencephalic leukoencephalopathy with subcortical cysts (MLC), a fatal genetic disorder marked by astrocytic edema. Mutations of several proteins cause this disease, but they occur primarily in MLC-1 [69], which is predominantly present in astrocytic endfeet. Brignone et al. employ a variety of techniques, including pull-down assays, electrophysiology and intracellular calcium ion imaging, to parse the pathway linking MLC-1 to edema. Using the glioblastoma cell line U251 transfected with wild type or mutant MLC-1, they demonstrate that MLC-1 potentiates the hypoosmotic-induced outward chloride current, and that this capacity requires phosphorylation of MLC-1 by Ca^2+^/Calmodulin-dependent protein kinase II (CaMKII). They also present evidence that influx of calcium ions into the cells, possibly through TRPV4 channels, is required for the activation of CaMKII. However, it does not do so directly, but instead through stimulation of calcium ion release from internal stores. Whether it is possible to manipulate this pathway to prevent or resolve cytotoxic edema for clinical benefit for MLC patients and for patients with other causes of cytotoxic edema remains to be determined.

Ischemia in the CNS results in accumulation of lactic acid and Na^+^ in astrocytes. Lactic acid accumulation, and hence acidosis, occurs from the anerobic metabolism of residual glucose and stored glycogen. Na^+^ accumulation occurs when the eventual depletion of these energy sources leads to decreased intracellular ATP, resulting in the failure of the Na^+^/K^+^-ATPase to pump Na^+^ from the cells. Both acidosis and Na^+^ accumulation have detrimental effects: acidosis can result in irreversible damage leading to necrosis [70], while Na^+^ accumulation inhibits glutamate uptake and increases Ca^2+^ uptake [71]. The astrocyte sodium-bicarbonate cotransporter 1 (NBCe1), which cotransports Na^+^ and HCO_3_^−^ across the plasma membrane, affects both these changes, but in opposite directions. In this Special Issue, Everaerts et al. [72] determined the direction of net flux through the astrocytic NBCe1 under ischemic conditions. They exposed hippocampal slice cultures from wild type mice or mice deficient for NBCe1 to a 2 min chemical ischemia, using 2-deoxyglucose to inhibit glycolysis and azide to inhibit oxidative phosphorylation. Assays with fluorescent indicators for Na^+^, pH and ATP revealed that NBCe1 operated in the forward mode under these conditions; that is, there was net import of Na^+^ and HCO_3_^−^ into the cell, ameliorating the ischemia-induced acidification, but exacerbating the increase in intracellular Na^+^. Awaiting further investigation is whether the same direction of net flux occurs during ischemia in animals, and whether manipulation of NBCe1 activity could be of clinical benefit in humans experiencing ischemia.

CD44 in a transmembrane glycoprotein that interacts with multiple binding partners at both its N-terminal extracellular domain and its C-terminal intracellular domain. A primary extracellular binding partner is hyaluronic acid, which is important for cell adhesion and perhaps has a role in axon guidance. Intracellular binding partners include several signaling proteins, such as Src kinase and focal adhesion kinase, which may regulate intracellular Ca^2+^ levels (reviewed in ref. [73]). Given its multiple binding partners and thus likely functions, it’s surprising that CD44 null mice are grossly normal; however, targeted analyses have uncovered defects in behavior, memory and regulation of inflammation. In the limited regions of the mature human CNS surveyed, CD44 has been found primarily on a subclass of astrocytes that reside in subpial and subependymal regions and extend long, unbranched, processes; it is not found on bushy, protoplasmic astrocytes. In this Special Issue, Al-Dalahmah et al. [74] confirm and extend these findings in a comprehensive analysis of the entire human CNS. Curiously, they find that in some CNS regions neurons are surrounded by CD44-positive astrocyte processes, whereas in other regions they are not. They also observe that although protoplasmic astrocytes do not express CD44 under normal conditions, they do in hypoxic and epileptic tissue from human patients and in vivo rat models of hypoxia and seizures. These findings provide basic information about CD44 expression in the CNS which should facilitate deciphering its functions.

In this Special Issue, Kruk et al. [2] investigated what functional ramifications epilepsy-induced CD44 expression might have by examining the effect of inhibiting astrocytic CD44 expression in the hippocampus of mice prior to kainate-induced seizures. Hippocampal-specific knockdown of CD44 was accomplished by injecting adeno-associated viruses expressing *cre* driven by a *GFAP* promoter into the hippocampus of mice carrying a floxed CD44 gene. A significant reduction in the number of behavioral seizures in the CD44 knockdown mice compared to controls was observed, as well as a reduction in astrocyte reactivity as measured by GFAP expression. Structural analyses revealed fewer contacts between astrocyte processes and synapses in the CD44 deficient mice compared to controls, and prevention of the loss of dendritic spines produced by KA-induced seizures that occurs in controls. These results, as well as others discussed in their paper, suggest CD44 as a therapeutic target for ameliorating seizures.

The findings of Kruk et al. [2] regarding CD44 implicate astrocytes as contributors to epileptogenesis. As reviewed by Bedner and Steinhäuser [1] in this Special Issue, astrocytes might also contribute to epileptogenesis through alterations in connexin proteins and the gap junctions they form. Relevant to epilepsy, gap junctions link groups of astrocytes to facilitate K^+^ buffering, which is critical for preventing the accumulation of K^+^ around active synapses that could lead to depolarization and hyperactivity. Consistent with K^+^ buffering being critical in epilepsy, gap junction connectivity is lost in animal models of temporal lobe epilepsy, and this loss precedes onset of behavioral seizures. Surprisingly, however, knockout of the primary astrocytic connexins, Cx30 and Cx43, does not cause seizures despite eliminating gap junction connectivity. Bedner and Steinhäuser explain that this likely reflects the multiple roles of connexins and gap junctions. Absence of some of these functions, like K^+^ buffering, could initiate or exacerbate seizures; whereas absence of others, like the provision of energy substrates to the neural network, could lower neuronal activity. The authors also review the possible relevance of connexin functions unrelated to gap junctions, including their formation of hemichannels that expose the astrocyte cytoplasm directly to the interstitial fluid. Their commentary on these complexities concludes with suggestions for determining causality, and for the potential for targeting connexins for epilepsy treatment.

In addition to TBI and ischemia, viral infection can be another source of chronic inflammation in the CNS. Many of the CNS viruses, including Zika, herpes, coronavirus and HIV, replicate in astrocytes [75,76]. In their review in this Special Issue, Potokar et al. [77] describe the particular properties of astrocytes that cause them to be welcoming viral hosts. These include their physical location in the CNS, metabolism, and array of viral receptors. Treatment of CNS viral infections will require appreciation of the role of astrocytes in their replication.

As noted above, inflammation accompanies many insults to the CNS, including TBI, ischemia, and viral infection. In addition to producing a direct deleterious effect on the injured tissue, inflammation can have an insidious indirect impact by disrupting the restorative effect of regeneration by differentiation of neural stem cells. In this Special Issue, Pavlou et al. [78] investigate the effects of inflammation on the differentiation of astrocytes from neural stem cells. Changes in gene expression and chromatin accessibility during the differentiation of astrocytes from murine neurospheres in the absence and presence of tumor necrosis factor-alpha (TNF) were compared. Their results indicate that the presence of TNF directs the stem cells along a reactive and incomplete astrocyte differentiation pathway, resulting in compromised astrocyte function. Transcription factors key to astrocyte differentiation in the presence and absence of TNF were also identified. These findings are paralleled by the restriction in chromatin accessibility that occurs during astrocyte differentiation being less extensive in the presence of TNF. Understanding the mechanisms and effects of inflammation on neural progenitor cell differentiation could lead to greater success for the use of neural progenitor cells to treat CNS disorders that are accompanied by inflammation.

Hyperammonemic encephalopathy is a clinical condition displaying both mental and motor manifestations. Coma occurs in its terminal stage, associated with fatal brain edema [79,80,81,82]. The disorder is defined by an elevated level of blood ammonia, most commonly caused by a deficiency in the urea cycle or liver failure, the latter being referred to as hepatic encephalopathy. In the brain, ammonia is metabolized by the astrocyte-specific enzyme glutamine synthetase, which joins ammonia to glutamate to form glutamine [83,84,85]. However, if CNS ammonia levels rise above the capacity of the glutamine synthetase to detoxify it, multiple astrocyte functions become compromised, including regulation of glutamate, K^+^, Na^+^, Ca^2+^ and pH, leading to death if unchecked [86,87,88,89,90]. In this Special Issue, Sepehrinezhad et al. [91] review another function of astrocytes that could contribute to the pathology of hepatic encephalopathy—its role in the glymphatic system. This system serves to remove potentially toxic metabolic products and misfolded proteins from the brain through the flow of CSF into and through the brain parenchyma to eventually drain into the lymph [92]. This flow is driven by the pressure difference between pulsating arterioles and the brain interstitial fluid, and is facilitated by water flow through astrocytic AQP4 channels. Sepehrinezhad et al. [91] review the evidence for reduced flow through the glymphatic system in hepatic encephalopathy. Among the possible causes are astrocytic swelling, which reduces the arterial/brain parenchyma pressure differential, and mislocalization of AQP4 channels from astrocytic endfeet to the cell body.

Alterations in astrocyte function and morphology are associated with multiple addictive disorders (reviewed in ref. [93]). In this Special Issue, Siemsen et al. [94] report observations of astrocytic changes associated with the remodeling of the connectivity of cortical prelimbic neurons to the nucleus accumbens that is considered responsible for relapse of heroin addiction. Previous studies from their lab and others have focused on the nucleus accumbens, where heroin self-administration in rats was found to produce downregulation of glutamate uptake by astrocytes and decreased contact between astrocyte processes and synapses, with the net result of increased glutamate spillover to perisynaptic targets. Administration of the antioxidant N-acetylcysteine prevented both of these astrocytic changes and drug-seeking relapse, suggesting a causal relationship [95,96,97]. In their present report, Siemsen et al. [94] performed similar studies of astrocytes at the cortical prelimbic end of the neuronal connection. Changes in astrocytes were also observed in this region, but differed from those in the nucleus accumbens—there was little if any reduction in glutamate uptake, and an increase, rather than a decrease, in the association of astrocytic processes with synapses. In addition, heroin self-administration resulted in the astrocytic processes becoming longer and more highly branched. All of these changes were prevented by treatment with N-acetylcysteine, raising the possibility that astrocyte remodeling in the prelimbic cortex as well as in the nucleus accumbens contributes to drug addiction.

Another pathway by which astrocytes could affect drug addiction is via their opioid receptors. Murlanova et al. [98] explore this possibility in this Special Issue by studying mice in which one of the alleles encoding the μ-opioid receptor 1 has been knocked out specifically in astrocytes. Several effects of this heterozygous knockout are noted, including a greater increase in the morphine-induced locomotor activity, a greater and longer-lived conditioned place aversion following morphine withdrawal, and an increased rate of oxygen consumption by astrocytes cultured from untreated or morphine-injected knockout mice compared to wild type controls. These findings suggest that the astrocyte μ-opioid receptor 1 serves to tamp down morphine-induced behaviors, perhaps by decreasing the rate of astrocytic oxidative phosphorylation.

Multiple studies have reported alterations of astrocytes in psychiatric disorders, but often with conflicting findings. In the Introduction to their paper in this Special Issue, Zhang et al. [99] summarize these prior observations and point out the variables that could cause the discrepancies, including heterogeneity among astrocytes and effects of treatment interventions. They then take these variables into account in an analysis of gene expression in several psychiatric diseases. For example, when the gene expression data from patients with schizophrenia or depression were compared to the expression profiles that define several astrocyte subgroups, the expression data matched the profiles of some astrocyte subgroups, but not others, indicating a role for these astrocyte subsets in the diseases. Proteins encoded by genes with increased expression identified biological pathways that might contribute to disease phenotype. Among these were several protein kinases, raising the possibility that their targeting could be therapeutic. As a cautionary note, experiments using rats to test the expression effects of psychotropic drugs under controlled conditions revealed discrepancies with data from humans, calling into question the use of rodent models to study human psychiatric disease.

Glaucoma is an insidious eye disorder affecting more than 2% of the world’s population aged 40 or older [100]. It involves damage to the optic nerve axons and death of retinal ganglion cells (RGCs) resulting in progressive reduction of the visual field. The primary cause is increased intraocular pressure (IOP) due to defective drainage of aqueous humor inside the eye. Death of the RGCs is believed to be caused by physical stress exerted by the IOP on their axons after they gather together to form the optic nerve. The region believed particularly sensitive to this stress is the astrocytic glial lamina, through which the optic nerve passes as it exits the eye. Since this region is primarily composed of astrocytes, and it contains an increased number of reactive astrocytes in glaucoma, astrocytes are considered a likely contributor to the disorder. In this Special Issue, Zhu et al. [101] use a mouse model of glaucoma to identify changes in astrocytes that are linked to glaucoma. They note that astrocytes in the glial lamina of older mice (40 weeks old) show some of the same changes found in younger mice (13 weeks old) treated to produce elevated IOP, yet do not have glaucoma. Thus, to sharpen their discrimination of changes specific to glaucoma, they compare gene expression profiles of their young glaucoma model mice to both age-matched and 40-week-old controls. Although about 80% of the expression differences between the young treated and untreated mice are also present in the comparison to normal older mice, the glaucoma mice uniquely increased expression of genes involved with process formation and extension. This suggested the possibility of increased phagocytosis of axonal mitochondria undergoing degeneration, a process normally present in the astrocytic glial lamina. Double fluorescent labeling of axonal mitochondria and astrocytes indeed revealed an increase in this activity. Surprisingly, for both this activity and gene expression, astrocytes with the same properties were observed in both the treated and untreated mice, with only the proportion of activated astrocytes increased. The authors discuss the possibility that this reflects two distinct but interconvertible populations of astrocytes, and that there might be clinical benefit to regulating their interconversion.

Together, the 22 papers in this Special Issue describe 22 different ways in which astrocytes are implicated in CNS disorders. This is probably just the tip of the iceberg, but a tip worth recognizing.
